# Cross Talk between Chemosensory Pathways That Modulate Chemotaxis and Biofilm Formation

**DOI:** 10.1128/mBio.02876-18

**Published:** 2019-02-26

**Authors:** Zhou Huang, Yun-Hao Wang, Hai-Zhen Zhu, Ekaterina P. Andrianova, Cheng-Ying Jiang, Defeng Li, Luyan Ma, Jie Feng, Zhi-Pei Liu, Hua Xiang, Igor B. Zhulin, Shuang-Jiang Liu

**Affiliations:** a State Key Laboratory of Microbial Resources and Environmental Microbiology, Research Center, Institute of Microbiology, Chinese Academy of Sciences, Beijing, China; b University of Chinese Academy of Sciences, Beijing, China; c Department of Microbiology, The Ohio State University, Columbus, Ohio, USA; Institut Pasteur; University of California, Santa Cruz; Dartmouth

**Keywords:** *Comamonas*, biofilms, chemoreceptors, chemotaxis, phosphotransfer, signal transduction

## Abstract

Complex chemosensory systems control multiple biological functions in bacteria, such as chemotaxis, gene regulation, and cell cycle progression. Many species contain more than one chemosensory system per genome, but little is known about their potential interplay. In this study, we reveal cross talk between two chemosensory pathways that modulate chemotaxis and biofilm formation in Comamonas testosteroni. We demonstrate that some chemoreceptors that govern chemotaxis also contribute to biofilm formation and these chemoreceptors can physically interact with components of both pathways. Finally, we show that the chemotaxis histidine kinase CheA can phosphorylate not only its cognate response regulator CheY_2_ but also one of the response regulators from the pathway mediating biofilm formation, FlmD. The phosphoryl group transfer from CheA to CheY_2_ is much faster than that from CheA to FlmD, which is consistent with chemotaxis being a fast response and biofilm formation being a much slower developmental process. We propose that cross talk between chemosensory pathways may play a role in coordination of complex behaviors in bacteria.

## Abstract (precis)

In many bacteria, two or more homologous chemosensory pathways control several cellular functions, such as motility and gene regulation, in response to changes in the cell’s microenvironment. Cross talk between signal transduction systems is poorly understood; while generally it is considered to be undesired, in some instances it might be beneficial for coregulation of complex behaviors. We demonstrate that several receptors from the pathway controlling motility can physically interact with downstream components of the pathway controlling biofilm formation. We further show that a kinase from the pathway controlling motility can also phosphorylate a response regulator from the pathway controlling biofilm formation. We propose that cross talk between two chemosensory pathways might be involved in coordination of two types of cell behavior—chemotaxis and biofilm formation.

## INTRODUCTION

Chemotaxis and biofilm formation are survival strategies that allow microorganisms to successfully find and dwell in environments. Chemotaxis is a process of active swimming toward attractants or away from repellents, which allows flagellated bacteria to monitor changes in the environment. Chemotaxis is best understood in model organisms Escherichia coli and Salmonella enterica (1). A chemotactic response is initiated by chemoreceptors, also called methyl-accepting chemotaxis proteins (MCPs) (2, 3) that detect various environmental signals through their sensory domains (4). Chemoreceptor signaling domains modulate the activity of the chemotaxis histidine kinase CheA. Following autophosphorylation, CheA transfers the phosphoryl group to the response regulator CheY. Phosphorylated CheY-P interacts with the flagellar switch protein FliM, causing a change in the direction of flagellar rotation (2, 5). Within the same chemotaxis pathway, the phosphatase CheZ (6), the methyltransferase CheR (7), and the methylesterase CheB (8) contribute to signal termination and adaptation.

In contrast to E. coli, which has only five chemoreceptors, many bacteria have a larger number of chemoreceptors; on average, fourteen chemoreceptor genes per bacterial genome were reported (9). In addition to chemotaxis, chemoreceptors and associated chemosensory pathways are implicated in regulation of twitching motility (10, 11), cell differentiation (12, 13) and aggregation (14), and biofilm formation (15–17). In Pseudomonas putida, the polyamine chemoreceptor McpU and the L-amino acids chemoreceptor McpA mediate chemotaxis and also contribute to biofilm formation (16).

Biofilm formation, a process of cell attachment and growing in aggregates on surfaces, is a regulated process that has been extensively investigated in model organisms, such as *Pseudomonas* species (18–22). In Pseudomonas aeruginosa, cyclic diguanosine-5′-monophosphate (c-di-GMP)-mediated signaling is the key regulatory circuit in biofilm formation. As a second messenger, c-di-GMP regulates biofilm formation by promoting the production of exopolysaccharides (23) and/or repressing synthesis of bacterial flagella (24). The Wsp chemosensory pathway in P. aeruginosa recognizes signals via its dedicated chemoreceptor WspA and contributes to biofilm formation via its response regulator WspR, which has diguanylate cyclase activity (15, 25). Together with other diguanylate cyclases and phosphodiesterases that modulate c-di-GMP levels as well as quorum sensing and small RNA signaling pathways, this chemosensory system contributes to a complex network that regulates biofilm formation (reviewed by Fazli et al. [20]).

Comamonas testosteroni CNB-1 belongs to a class of betaproteobacteria; it was isolated from a wastewater treatment bioreactor and grows on organic acids and aromatic compounds (26, 27). C. testosteroni is studied primarily as a promising organism for bioremediation of organics-contaminated environments: it forms organic-pollutant-degrading biofilms in natural ecosystems and water treatment systems (28). The process and mechanisms of biofilm formation in *C. testosteroni* are not well understood. *C. testosteroni* CNB-1 genome contains one chemotaxis (*che*) gene cluster and one chemotaxis-like (*flm*) gene cluster and nineteen chemoreceptor genes (29). In this study, we show that the *flm* cluster is involved in modulating biofilm formation in *C. testosteroni* and identify the FlmD protein as a response regulator for this behavior. We demonstrate that seven chemoreceptors contribute to biofilm formation, including those that are known to mediate chemotaxis. We also demonstrate that the CheA kinase can phosphorylate the FlmD response regulator, albeit at a much lower rate than its cognate response regulator CheY_2_. Therefore, we propose that chemotaxis and biofilm formation could be coregulated by the interplay between Che and Flm chemosensory pathways in *C. testosteroni*. Cross talk between chemosensory pathways in many other bacteria might play a similar role in coregulation of these and other types of cell behavior.

## RESULTS

### Two chemosensory pathways modulate the chemotactic response and biofilm formation in *C. testosteroni*.

**FIG 1:**
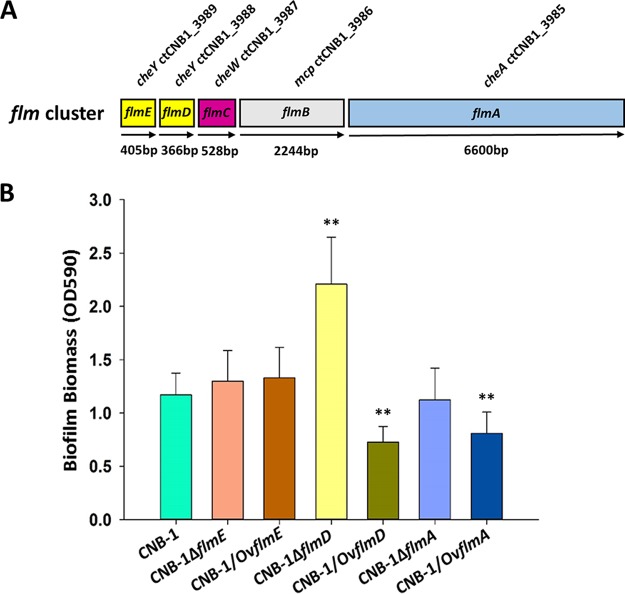
Flm pathway regulates biofilm formation. (A) Diagram of the *flm* genetic cluster. (B) Biofilm formation by *flm* gene deletion and overexpression mutants measured by a crystal violet assay. Data are the mean values plus standard deviations from triplicates. Values that are significantly different by Student’s *t* test are indicated by asterisks as follows: **, *P* < 0.01.

Analysis of the *C. testosteroni* CNB-2 (identical to CNB-1 except for the loss of the pCNB plasmid) complete genome using the MiST.2 database (30) revealed two genetic clusters (Fig. 1; see also Fig. S1 in the supplemental material) encoding chemosensory pathways. On the basis of the results obtained in this study, we termed them *che* and *flm* clusters. The *che* cluster contained a complete set of genes coding for chemotaxis proteins, including the flagellar motor switch components (FliG, FliM, and FliN), the histidine kinase CheA, two response regulators CheY_1_ and CheY_2_, the phosphatase CheZ, the adaptor protein CheW, the methyltransferase CheR, the methylesterase CheB, and the deamidase CheD. Matching CheA, CheW, CheB, and CheR sequences to hidden Markov models designed for specific chemotaxis pathways revealed that the pathway encoded by the *che* cluster belongs to the evolutionary class F7 (31). The best-studied chemotaxis pathway in the model organism E. coli belongs to this class. Consequently, this pathway in *C. testosteroni* was also predicted to mediate chemotaxis. We have previously deleted the *cheA* gene (32), and in-frame deletions of *cheW* and *cheY* genes were made in this study. All the mutants were characterized for chemotaxis, and results are shown in Fig. S1B. As expected, CheA and CheW were essential for chemotaxis in strain CNB-1. Two response regulators, CheY_1_ and CheY_2_, are encoded in the *che* gene cluster. Deletion of *cheY*_2_ resulted in a complete loss of the chemotactic response, while the deletion of *cheY*_1_ only partly reduced chemotaxis (Fig. S1B). We also found that CheA and CheY_2_ from strain CNB-1 were able to restore chemotactic response in the corresponding E. coli mutants (Fig. S1C).

10.1128/mBio.02876-18.1 FIG S1 Che pathway regulates chemotaxis. (A) Diagram of the *che* genetic cluster. (B and C) Functional characterization of the *che* cluster in *C. testosteroni* (B) and *E. coli* (C) by swimming plate. Shown are the means and standard deviations from three independent experiments conducted in triplicate. Download FIG S1, TIF file, 0.4 MB. Copyright © 2019 Huang et al. 2019 Huang et al. This content is distributed under the terms of the Creative Commons Attribution 4.0 International license.

The *flm* gene cluster had not been studied previously. It contains genes encoding a CheA-like histidine kinase (termed FlmA), a CheW-like adaptor protein (termed FlmC), a chemoreceptor (ctCNB1_3986, termed FlmB), and two CheY-like response regulators that contain the conserved aspartyl residue, which serves as the phosphor-acceptor site (termed FlmD and FlmE). Matching FlmA (CheA-like) and FlmC (CheW-like) sequences to hidden Markov models designed for specific chemotaxis pathways revealed that the pathway encoded by the *flm* cluster belongs to the evolutionary class Tfp (31), named after type IV pilus-mediated motility. The Tfp pathway in P. aeruginosa (also known as Chp/Pil) regulates twitching motility (11) and causes alterations in the cAMP levels (33). Orthologous relationships between Chp/Pil and Flm were established by showing that ChpA-FlmA, PilJ-FlmB, PilI-FlmC, PilH-FlmD, and PilG-FlmE are mutual best BLAST hits when searched against the respective genomes (see Table S1 in the supplemental material). Although Flm is orthologous to Chp/Pil, it lacks MCP-modifying enzymes CheB and CheR (searches with Tfp-specific CheB and CheR sequences failed to identify any homologs in the *C. testosteroni* genome), and FlmB, a chemoreceptor associated with this pathway, lacks methylation sites (Fig. S3 and Table S2) that are conserved in its P. aeruginosa ortholog PilJ (34).

10.1128/mBio.02876-18.7 TABLE S1 Orthologous relationships between components of the Flm and Chp/Pil pathways. Results of mutual best BLAST hits against corresponding genomes are shown along with their E values, percentage of identity, and length coverage. Download Table S1, PDF file, 0.01 MB. Copyright © 2019 Huang et al. 2019 Huang et al. This content is distributed under the terms of the Creative Commons Attribution 4.0 International license.

10.1128/mBio.02876-18.8 TABLE S2 Chemosensory apparatus of *C. testosteroni* CNB-2. Chemotaxis class definition for chemoreceptors is according to Alexander and Zhulin (35), where the number of helical heptads in signaling domain defines the class. Chemotaxis class definition for other proteins is according to Wuichet and Zhulin (31). Conserved methylation sites (M1 through M4) and a CheB/CheR-binding pentapeptide are deduced from the multiple sequence alignment shown in Fig. S3. Download Table S2, PDF file, 0.04 MB. Copyright © 2019 Huang et al. 2019 Huang et al. This content is distributed under the terms of the Creative Commons Attribution 4.0 International license.

While the Chp pathway in P. aeruginosa regulates twitching motility, we did not detect twitching motility in *C. testosteroni* strain CNB-1 under any condition tested. The Flm pathway had no effect on chemotaxis (Fig. S1D). The wild-type strain CNB-1 cells form a pellicle biofilm at the boundary of medium and air when grown in broth, and we tested whether the Flm pathway is involved in biofilm formation. We found that deletion of *flmD* resulted in a significant increase in biofilm formation (Fig. 1B), which suggested that FlmD functioned as a negative response regulator. We also observed that overexpression of the kinase FlmA and the response regulator FlmD resulted in a significant reduction of biofilm formation (Fig. 1B). Neither deletion nor overexpression of *flmE* (coding for another response regulator) had a significant effect on biofilm formation (Fig. 1B).

### Multiple chemoreceptors modulate biofilm formation in *C. testosteroni*.

**FIG 2:**
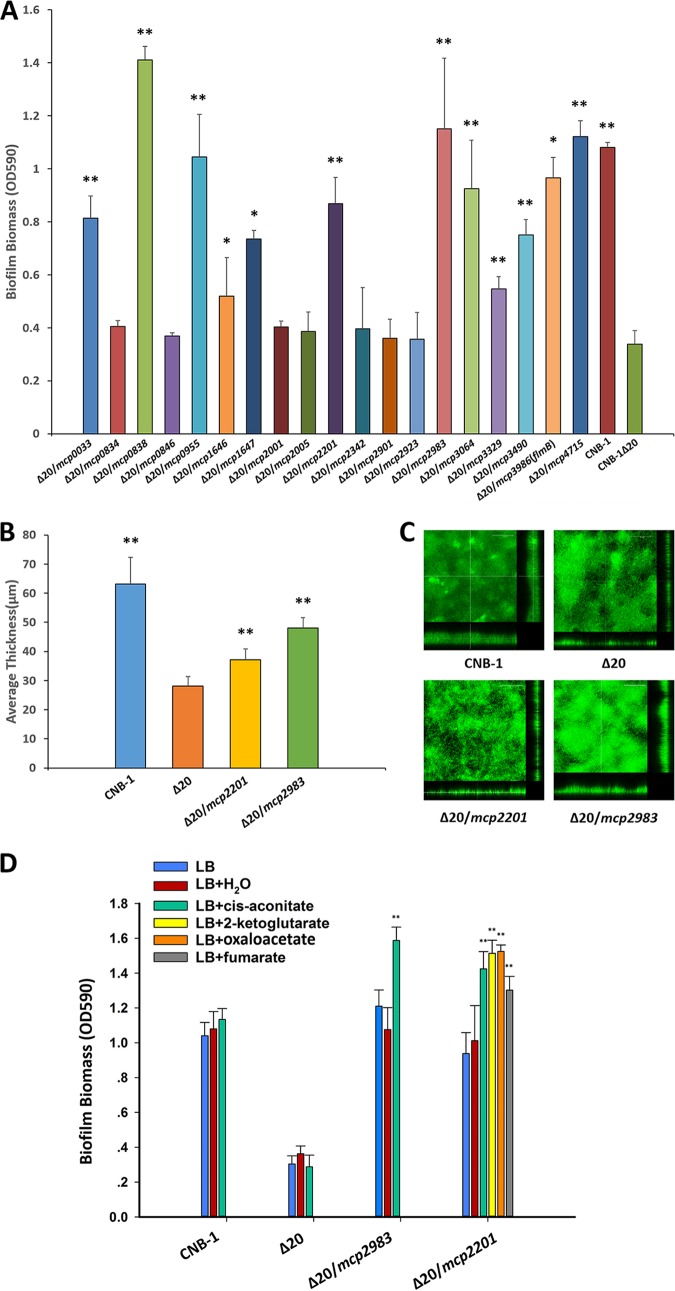
Chemoreceptors are involved in biofilm formation. (A) Biofilm formation by the CNB-1Δ20 mutant complemented with individual chemoreceptor genes measured by a crystal violet assay. (B) Average biofilm thickness by strain CNB-1, CNB-1Δ20, and chemoreceptor-complemented strains, calculated from confocal images. (C) Representative images of biofilm by confocal laser scanning microscopy (front view, *x*-axis profile, and *y*-axis profile). (D) Biofilm formation in the presence and absence of MCP2983 and MCP2201 ligands (final concentration, 2 mM). The values are means plus standard deviations from three independent experiments conducted in triplicate. Values that are significantly different are indicated by asterisks as follows: *, *P* < 0.05 by Student’s *t* test or rank sum test; **, *P* < 0.01 by Student’s *t* test or rank sum test.

The early draft of *C. testosteroni* genome listed 20 chemoreceptor genes (reflected in the name of the chemoreceptor-null mutant CNB-1Δ20). The latest, high-quality whole genome contains 19 chemoreceptors that have diverse domain architectures (Fig. S2). Using previously described hidden Markov models (35), we assigned FlmB (MCP3986) to the 40H class (contains 40 helical heptads in the cytoplasmic signaling domain) and all other chemoreceptors (except for MCP0846, which did not match confidently to any model) to the 36H class (contains 36 helical heptads in the cytoplasmic signaling domain) (Table S2 and Fig. S3). While FlmB (MCP3986) is an ortholog of PilJ and it is predicted to interact with the Flm pathway, the 36H class chemoreceptors are known and predicted to interact with the F7 chemotaxis class (31, 34), i.e., the Che pathway in *C. testosteroni*. We analyzed the biofilm formation abilities of *C. testosteroni* CNB-1 mutants deficient in chemoreceptor genes. Deletions of individual chemoreceptor genes did not result in significant changes in biofilm formation (Fig. S4): however, the chemoreceptor-null mutant CNB-1Δ20 was severely affected in biofilm formation (Fig. 2), while its growth rate was not affected (Fig. S5). We then complemented the CNB-1Δ20 mutant with each of the 19 chemoreceptor genes, and the biofilm formation was assessed by using crystal violet staining. Unexpectedly, not only FlmB (MCP3986) but also six other chemoreceptors, namely, MCP0838, MCP0955, MCP2201, MCP2983, MCP3064, and MCP4715, restored biofilm formation to at least 80% of the wild-type CNB-1 (Fig. 2A). MCP2201 and MCP2983 were previously identified as chemoreceptors for chemotaxis (32, 36). Using confocal laser scanning microscopy, we showed that not only the adhesion ability but also the pellicle formation was restored by MCP2201 and MCP2983 (Fig. 2B and C). We tested further whether the ligands that are recognized by these chemoreceptors and trigger chemotactic responses would also affect biofilm formation. The addition of *cis*-aconitate, which is the sole ligand for MCP2983 (36), resulted in a significant increase in biofilm formation. Similarly, 2-ketoglutarate, *cis*-aconitate, fumarate, and oxaloacetate that are known ligands for MCP2201 (32) significantly promoted biofilm formation (Fig. 2D). These effects were seen only in the presence of the corresponding chemoreceptors (Fig. 2D), and the presence of a ligand did not significantly alter cell growth (Fig. S6).

10.1128/mBio.02876-18.2 FIG S2 Domain architectures of *C. testosteroni* chemoreceptors revealed by using CDvist. Transmembrane regions and Pfam domains are shown. Download FIG S2, TIF file, 1.3 MB. Copyright © 2019 Huang et al. 2019 Huang et al. This content is distributed under the terms of the Creative Commons Attribution 4.0 International license.

10.1128/mBio.02876-18.3 FIG S3 Multiple sequence alignment of signaling domains from *C. testosteroni* chemoreceptors. Predicted methylation sites are shown in red, and adjucent consensus sequences are shown in gray; predicted C-terminal pentapeptides are highlighted in yellow. Download FIG S3, PDF file, 0.06 MB. Copyright © 2019 Huang et al. 2019 Huang et al. This content is distributed under the terms of the Creative Commons Attribution 4.0 International license.

10.1128/mBio.02876-18.4 FIG S4 Single chemoreceptor deletion does not affect the biofilm formation. Biofilm formation by each single chemoreceptor mutant measured by a crystal violet assay. Download FIG S4, PDF file, 0.5 MB. Copyright © 2019 Huang et al. 2019 Huang et al. This content is distributed under the terms of the Creative Commons Attribution 4.0 International license.

10.1128/mBio.02876-18.5 FIG S5 Growth rates of the receptor-free mutant (Δ20) and the wild type (CNB-1) on different carbon sources. Gen, gentisate; 4HB, 4-hydroxybenzoate; 3HB, 3-hydroxybenzoate; PCA, protocatechuate. Download FIG S5, TIF file, 2.0 MB. Copyright © 2019 Huang et al. 2019 Huang et al. This content is distributed under the terms of the Creative Commons Attribution 4.0 International license.

10.1128/mBio.02876-18.6 FIG S6 Growth of chemoreceptor mutants in the presence and absence of their ligand *cis*-aconitate. CNB-1, wild type; Δ20/*mcp2201*, Δ20 mutant complemented with the corresponding chemoreceptor gene; Δ20/*mcp2983*, Δ20 mutant complemented with the corresponding chemoreceptor gene; Δ20, mutant lacking all chemoreceptor genes. Growth conditions are described in Materials and Methods. Download FIG S6, PDF file, 1.2 MB. Copyright © 2019 Huang et al. 2019 Huang et al. This content is distributed under the terms of the Creative Commons Attribution 4.0 International license.

### Physical interactions between the Che and Flm proteins.

**FIG 3:**
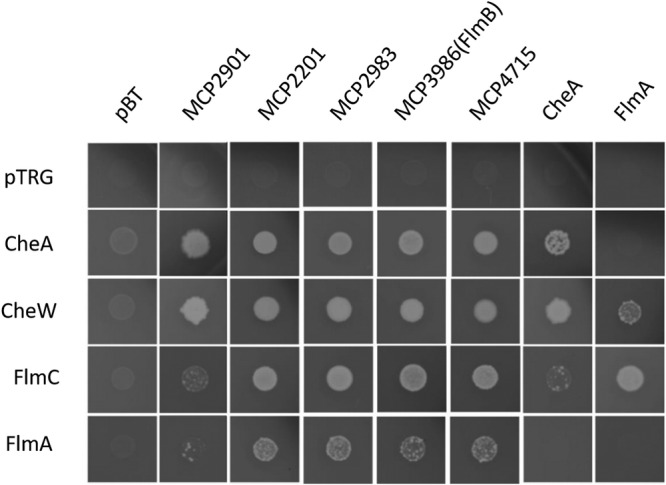
Chemoreceptors interact with other components of Che and Flm pathways. The growth of bacterial two-hybrid system cotransformants is shown on selective screening medium plates. Better growth represents a stronger interaction.

The observation that MCP2201 and MCP2983 that are known to mediate chemotaxis also affected biofilm formation suggested there could be potential cross talk between Che and Flm pathways. We used bacterial two-hybrid systems (BACTH) to identify possible protein-protein interactions between the components of the two pathways (Fig. 3). As expected, interactions between 36H class chemoreceptors (MCP2201, MCP2901, MCP2983, and MCP4715) and the histidine kinase CheA and the adaptor protein CheW were observed. Unexpectedly, we also observed interactions of these chemoreceptors with the kinase FlmA and the adaptor FlmC (Fig. 3), which suggests that physical interactions of chemoreceptors from the Che pathway with a kinase and an adaptor from the Flm pathway might be one of the potential mechanisms for observed cross talk.

### Phosphotransfer between CheA and response regulators of the Che and Flm pathways.

**FIG 4:**
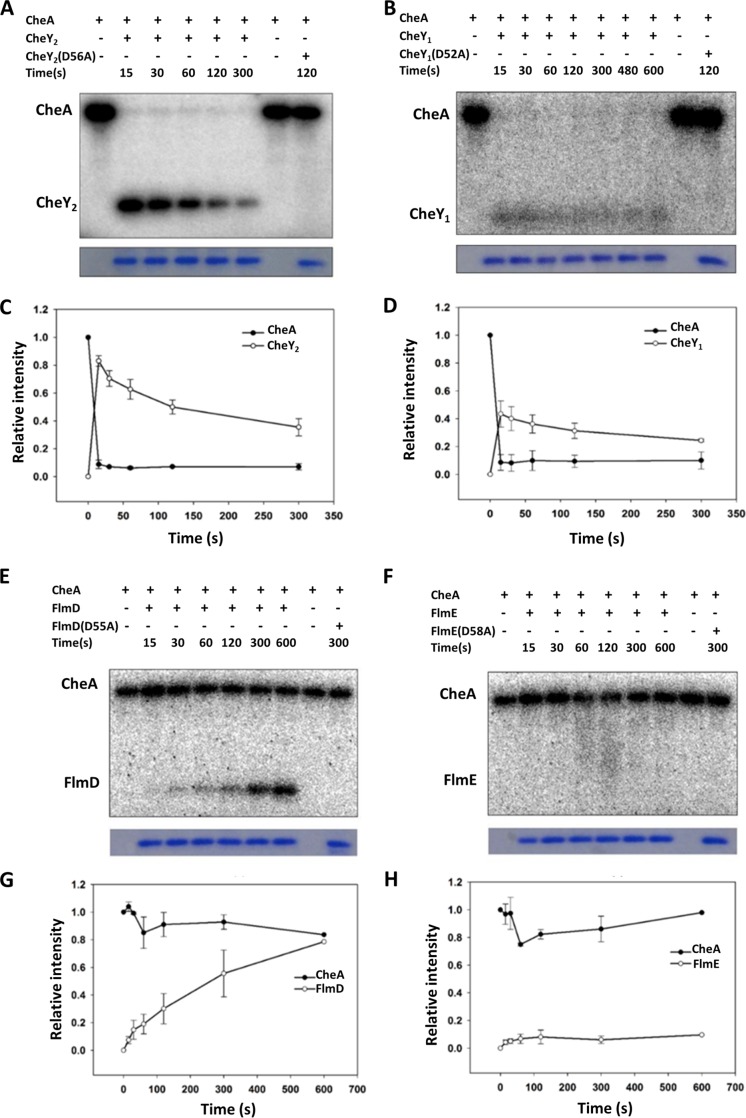
The phosphoryl group transfers from CheA to CheY_1_, CheY_2_, and FlmD. (A to H) Representative phosphotransfer images (A, B, E, and F) and time courses of the phosphotransfer from CheA-P to CheY_1_, CheY_2_, FlmD, and FlmE (C, D, G, and H). The data are presented as the mean values of three independent experiments. Error bars represent the standard deviations (SD).

The phosphotransfer from the histidine kinase CheA to the response regulator CheY is the first step in signal transduction during chemotaxis. In order to measure phosphotransfer between kinases and response regulators of the two pathways, we purified recombinant CheA, CheY_1_, and CheY_2_, as well as FlmD and FlmE. Efforts to purify the histidine kinase FlmA were unsuccessful. As expected, we recorded a strong and clear phosphotransfer from CheA to one of the chemotaxis response regulators, CheY_2_ (Fig. 4A). We also observed a phosphotransfer from CheA to CheY_1_, but with much faster CheY autodephosphorylation rate (Fig. 4C and D), which implied that CheY_1_ might play a role as a phosphate sink, as previously suggested for other chemotaxis pathways with two CheY response regulators (37, 38). Unexpectedly, we observed that the kinase CheA phosphorylated FlmD (but not another response regulator, FlmE) (Fig. 4E and F). Compared to CheY_2_, the phosphotransfer from CheA to FlmD occurred at a significantly lower rate. FlmD received an equivalent level of phosphorylation from CheA at 600 s, while CheY_2_ achieved it at 15 s (Fig. 4C and G). CheY_2_ was quickly phosphorylated by CheA, and the phosphorylated CheY_2_ decayed after 15 s (Fig. 4A). FlmD was continuously phosphorylated even after 600 s of incubation.

The conserved aspartate residues serving as phosphorylation sites (39) were unchanged in all three response regulators: D52 in CheY_1_, D56 in CheY_2_, and D55 in FlmD. When the phosphorylation site was mutated from aspartate to alanine, the phosphotransfer from CheA to each mutant response regulator was no longer observed (Fig. 4).

### FlmD modulates biofilm formation in the presence and absence of FlmA.

**FIG 5:**
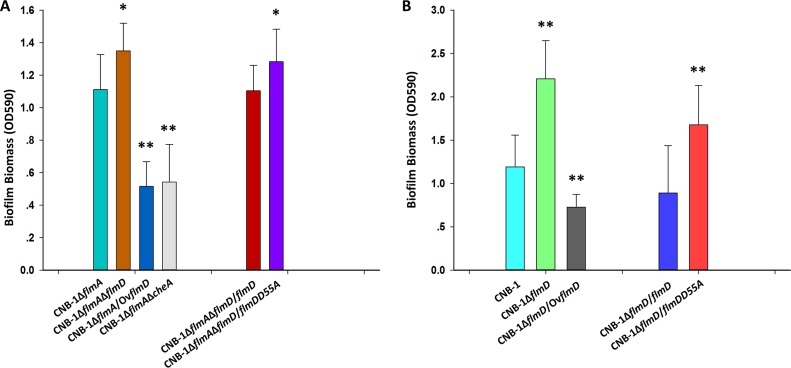
FlmD regulates biofilm formation *in vivo* in the presence and absence of FlmA. (A) Functional characterization of FlmD mutants in the absence of FlmA (A) and in the presence of FlmA (B) measured by crystal violet assay. Shown are the means and standard deviations from three independent experiments conducted in triplicate. *, *P* < 0.05 by Student’s *t* test or rank sum test; **, *P* < 0.01 by Student’s *t* test or rank sum test.

On the basis of the observation that kinase CheA can phosphorylate the response regulator FlmD (potential signal cross talk between Che and Flm pathways), we carried out experiments using mutants to demonstrate *in vivo* that such interplay might affect biofilm formation. To exclude the influence from the kinase FlmA (whose cognate target is FlmD/FlmE) on the potential phosphotransfer from CheA to the response regulator FlmD, we used the *flmA* knockout mutant. As shown in Fig. 5A, deletion of *flmD* resulted in upregulation of biofilm formation, whereas overexpression of FlmD resulted in significant reduction of biofilm formation, and deletion of *cheA* also caused a significant decrease in biofilm formation. These results demonstrated that the response regulator FlmD and the kinase CheA modulate biofilm formation in the absence of the FlmA kinase. This was further confirmed by the fact that the D55A FlmD mutant deficient in the phosphor-acceptor site showed enhanced biofilm formation compared to the FlmA mutant and the wild type (Fig. 5A and B). On the basis of these results, we conclude that a phosphorylated FlmD negatively regulates biofilm formation. This further supported by experiments showing that in the presence of the kinase FlmA, the phosphorylated response regulator FlmD (overexpressed as wild-type FlmD) also negatively regulates biofilm formation (Fig. 5B).

## DISCUSSION

Chemotaxis and biofilm formation are processes that are important for different lifestyles in bacteria. Chemotaxis is a rapid response to fluctuating conditions in the microenvironment, such as gradients of nutrients. Biofilm formation is a response to persistent changes, such as transition from a liquid environment to a surface. Actively moving chemotactic cells live in a planktonic state, whereas cells in biofilms live in a sessile state. Switching from one lifestyle to another requires coordinated regulation, and cross talk between regulatory systems might be one type of such coordination. We found that the same signals—organic acids—serve as chemoattractants and stimulate biofilm formation, which might seem counterintuitive, because typically, these are inversely regulated processes. One possible explanation is that chemotaxis allows *C. testosteroni* cells to detect low concentrations of organic acids and by moving along their gradients to find higher concentrations that sustain metabolism and proliferation and trigger biofilm formation. In such a scenario, biofilm helps bacteria to establish themselves and to remain in a favorable microenvironment.

Cross talk between chemotaxis and other signaling pathways, such as pili-mediated surface motility or virulence induction signaling system, has been proposed (40, 41) but not demonstrated. In this study, we showed that two chemosensory pathways in *C. testosteroni* modulate chemotaxis and biofilm formation. Comparative genomic analysis revealed that the *che* pathway in *C. testosteroni* belongs to the most abundant type of the chemotaxis signal transduction class, F7, which controls flagellar motility in a closely related model organism E. coli. Seventeen chemoreceptors from the *C. testosteroni* genome were predicted computationally to feed into the Che pathway, and three of them were previously shown to govern chemotaxis (32, 36, 42). By showing that the chemotaxis response was lost in *cheA* (32), *cheW,* and *cheY* (this study) mutants, we firmly established the role for this signal transduction pathway.

Computational analysis showed that the second chemosensory pathway in *C. testosteroni*, which we termed Flm, belongs to the evolutionary class Tfp (31), and it is orthologous to the Chp/Pil pathway, which modulates twitching motility and virulence in P. aeruginosa (11, 33, 43). In comparison with Chp/Pil, the Flm pathway lacks three components: the additional adaptor protein ChpC, the methyltransferase CheR, and the methylesterase CheB. Furthermore, FlmB, the only chemoreceptor predicted to feed into the Flm pathway, lacks methylation sites, which is consistent with the loss of methylation/demethylation enzymes. Therefore, Flm function was expected to be somewhat different from that of the Chp/Pil pathway. We have found that the Flm pathway modulates biofilm formation in *C. testosteroni* and that the response regulator FlmD, which is the preferred target of FlmA kinase phosphorylation, serves as a negative response regulator. FlmD is orthologous to the response regulator PilH of the Chp/Pil pathway, which is also the preferred target of ChpA phosphorylation (44), but its function is not well understood. PilG, another response regulator of the Chp/Pil pathway, is required for Tfp function as a motility organelle and mechanosensor in P. aeruginosa (33, 45). We did not detect twitching motility in *C. testosteroni* under any condition tested, but we showed that FlmE, the PilG ortholog, has no role in biofilm formation. We searched for CyaA or CyaB homologs in the *C. testosteroni* genome that would potentially suggest that the Flm pathway might regulate cAMP levels, as does the orthologous pathway in P. aeruginosa (33); however, these searches failed to identify any proteins orthologous to CyaA and CyaB.

A chemosensory pathway modulating biofilm formation (namely, Wsp) was previously identified in P. aeruginosa (15, 25) and P. putida (16). The response regulator WspR contains a *c*-di-GMP cyclase domain (GGDEF) as the pathway output. As a result of chemosensory signal transduction, increased levels of *c*-di-GMP enhance biofilm formation (15, 25). The Wsp pathway belongs to a different evolutionary class—ACF (named ACF for alternative cellular functions) (31), and the Flm response regulators do not contain GGDEF domains. Furthermore, they are both comprised of a single response regulator receiver domain, similar to the chemotaxis response regulator CheY. Such single domain response regulators are ubiquitous, and they might have multiple, spatially separated targets (46). Biofilm formation is a very complex process (47), and the target for the response regulator FlmD remains to be identified.

**FIG 6:**
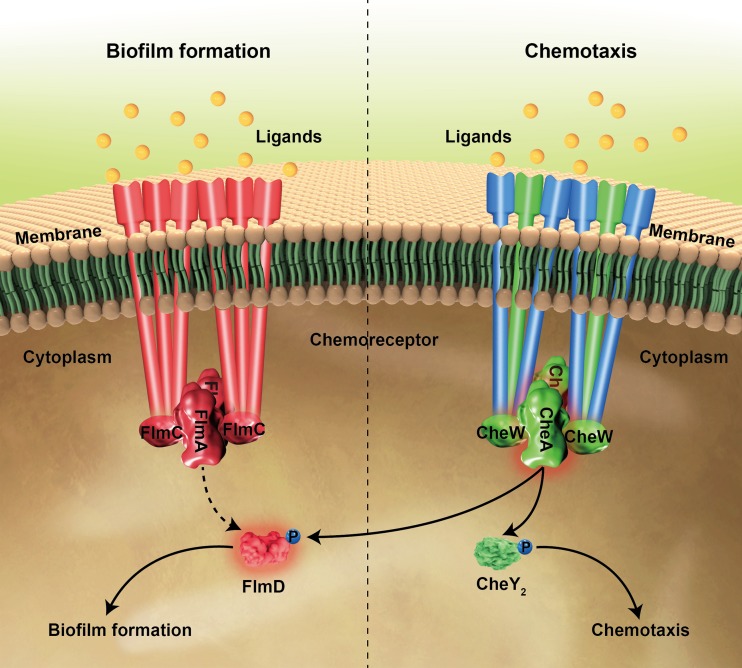
Model of signal transduction and cross talk between Che and Flm pathways. Proteins that are involved in chemotaxis only are shown in green, proteins that are involved in biofilm formation only are shown in red, and proteins that are involved in both chemotaxis and biofilm formation are shown in blue. Che and Flm pathways regulate chemotaxis and biofilm formation, respectively. Che pathway might also influence biofilm formation through the phosphotransfer from CheA to FlmD. The pathways that have not been genetically or biochemically confirmed are represented by dashed lines.

We documented cross talk between the two chemosensory systems in *C. testosteroni* at two potential sites (Fig. 6): (i) chemoreceptor interaction with a nonpartner pathway and (ii) phosphotransfer from a kinase to a nonpartner response regulator. Results obtained in bacterial two-hybrid screens raise the possibility that chemoreceptors from the Che pathway may interact with the adaptor protein FlmC and the histidine kinase FlmA. These interactions might be insignificant *in vivo*, because chemoreceptors from Che and Flm pathways belong to different length classes. Chemoreceptors of different length classes in orthologous systems in P. aeruginosa were found to possess pathway specificity determinants (34) that likely target them to “preferred” partners in spatially separated signaling arrays.

Phosphotransfer from the histidine kinase CheA to the response regulator FlmD is a more likely site for cross talk. Our results supporting FlmD phosphorylation by CheA (both *in vitro* and *in vivo*) were obtained in the absence of the histidine kinase FlmA. Similar cases of phosphotransfer from a histidine kinase to a noncognate response regulator in the absence of its own histidine kinase have been reported previously (48). However, a series of studies argue that such cross talk is physiologically irrelevant in wild-type cells *in vivo* (49–51). One of the key arguments is that in systems where cross talk was observed, the output of the system is blind to input stimulus (49, 52). In our case, the FlmD modulated output, i.e., biofilm formation, appears to be responsive to input stimulus: ligands specific to a chemoreceptor, which signals through CheA, modulated biofilm formation, and it was observed only in the presence of a corresponding chemoreceptor. Observations consistent with the proposition that components of chemosensory pathways controlling chemotaxis also modulate biofilm formation have been previously reported. The BdlA chemoreceptor in P. aeruginosa, which is predicted to feed into the chemotaxis pathway (34), is essential for biofilm dispersal (53, 54). Inactivation of the chemotaxis methyltransferase CheR (55) and the response regulator CheY (56) in P. aeruginosa also led to defects in biofilm formation, although the basis for this behavior is unknown. In a closely related bacterium Shewanella oneidensis, a chemosensory pathway was also implicated in biofilm formation (57), likely via the interaction of its response regulator CheY3 with the c-di-GMP-binding protein (58). The results described here provide potential mechanisms for these and other observations linking chemotaxis and biofilm formation and suggesting their coregulation.

## MATERIALS AND METHODS

### Bacterial strains and plasmids.

The bacterial strains and plasmids used in this study are listed in Table 1. *C. testosteroni* and its mutants were cultivated and maintained at 30°C in LB broth or on LB plates with 1.5% (wt/vol) agar; antibiotic (200 μg/ml kanamycin) was added when necessary. For E. coli, strains were grown at 37°C in LB, and kanamycin was used at 50 μg/ml when needed. Genetic disruption and complementation in *C. testosteroni* CNB-1 were conducted using pK18mobSacB and pBBR1MCS-2, respectively. The plasmids for overexpression were pBBR1MCS-2 derivative (pBBR1MCS2pfer) whose promoter was replaced with a strong promoter from *C. testosteroni* (Table 1).

**TABLE 1:** Bacterial strains and plasmids used in this study

Strain or plasmid	Relevant genotype and/or description	Reference or source
Comamonas testosteroni strains		
CNB-1		26
CNB-1Δ20	All putative chemoreceptor genes were disrupted in CNB-1	31
CNB-1ΔcheY_1_	CheY_1_(CtCNB1_0474) disrupted in CNB-1	This work
CNB-1ΔcheY_2_	CheY_2_(CtCNB1_0455) disrupted in CNB-1	This work
CNB-1ΔcheA	CheA(CtCNB1_0475) disrupted in CNB-1	31
CNB-1ΔcheW	CheW(CtCNB1_0476) disrupted in CNB-1	This work
CNB-1ΔflmA	FlmA(CtCNB1_3985) disrupted in CNB-1	This work
CNB-1ΔflmD	FlmD(CtCNB1_3988) disrupted in CNB-1	This work
CNB-1ΔflmE	FlmE(CtCNB1_3989) disrupted in CNB-1	This work
CNB-1ΔflmAΔflmD	FlmA FlmD double disruptions in CNB-1	This work
CNB-1ΔflmAΔcheA	FlmA CheA double disruptions in CNB-1	This work
Escherichia coli strains		
DH5α	F^−^ φ80d *lacZ*ΔM15 Δ(*lacZYA*-*argF*)*U169 recA1 endA1 hsdR17*(r_K_^−^ m_K_^+^) *supE44* λ^−^ *thi-1 gyrA96* * relA1 phoA*; host for DNA manipulations	TransGen
BL21(DE3)	F^−^ *ompT hsdS*(r_B_^−^ m_B_^−^) *gal dcm* (DE3)	Novagen
RP9535	CheA disrupted in E. coli RP437	Parkinson’s lab
RP5232	CheY disrupted in E. coli RP437	Parkinson’s lab

Plasmids		
pBBR1MCS-2	Km^r^; *lacPOZ*′ broad-host vector with R-type conjugative origin	66
pBBR1MCS2-mcp0033	Carries *mcp0033* to generate complementation	This work
pBBR1MCS2-mcp0034	Carries *mcp0834* to generate complementation	This work
pBBR1MCS2-mcp0838	Carries *mcp0838* to generate complementation	This work
pBBR1MCS2-mcp0846	Carries *mcp0846* to generate complementation	This work
pBBR1MCS2-mcp0955	Carries *mcp0955* to generate complementation	This work
pBBR1MCS2-mcp1646	Carries *mcp1646* to generate complementation	This work
pBBR1MCS2-mcp1647	Carries *mcp1647* to generate complementation	This work
pBBR1MCS2-mcp2001	Carries *mcp2001* to generate complementation	This work
pBBR1MCS2-mcp2005	Carries *mcp2005* to generate complementation	This work
pBBR1MCS2-mcp2201	Carries *mcp2201* to generate complementation	31
pBBR1MCS2-mcp2342	Carries *mcp2342* to generate complementation	This work
pBBR1MCS2-mcp2901	Carries *mcp*2901 to generate complementation	41
pBBR1MCS2-mcp2923	Carries *mcp2923* to generate complementation	This work
pBBR1MCS2-mcp2983	Carries *mcp2983* to generate complementation	35
pBBR1MCS2-mcp3064	Carries *mcp3064* to generate complementation	This work
pBBR1MCS2-mcp3329	Carries *mcp3329* to generate complementation	This work
pBBR1MCS2-mcp3986	Carries *mcp3986* to generate complementation	This work
pBBR1MCS2-mcp4715	Carries *mcp4715* to generate complementation	This work
pBBR1MCS2-flmD	Carries *flmD* to generate complementation	This work
pBBR1MCS2-flmDD55A	A mutation from an aspartate to an alanine in 55th residue	This work
pBBR1MCS2-flmE	Carries *flmE* to generate complementation	This work
pBBR1MCS2-flmED58A	A mutation from an aspartate to an alanine in 58th residue	This work
pBBR1MCS2pfer	Adds a strong constitutive promoter in pBBR1MCS-2	Our lab
pBBR1MCS2pfer-flmA	Carries *flmA* to overexpression	This work
pBBR1MCS2pfer-flmD	Carries *flmD* to overexpression	This work
pBBR1MCS2pfer-flmE	Carries *flmE* to overexpression	This work
pET28a-cheA	pET28a derivative for expression of CheA	41
pET28a-cheY_1_	pET28a derivative for expression of CheY_1_	This work
pET28a-cheY_1_(D52A)	pET28a derivative for expression of CheY_1_ with D52A mutation	This work
pET28a-cheY_2_	pET28a derivative for expression of CheY_2_	This work
pET28a-cheY_2_(D56A)	pET28a derivative for expression of CheY_2_ with D56A mutation	This work
pET28a-flmD	pET28a derivative for expression of FlmD	This work
pET28a-flmD(D52A)	pET28a derivative for expression of FlmD with D52A mutation	This work
pET28a-flmE	pET28a derivative for expression of FlmE	This work
pET28a-flmE(D58A)	pET28a derivative for expression of FlmE with D58A mutation	This work
pBT	Bacterial two-hybrid bait plasmid with λ repressor protein (λcI)	Stratagene
pBT-cheA	pBT derivative with λcl linked to the N-terminal region of CheA	This work
pBT-flmA	pBT derivative with λcl linked to the N-terminal region of FlmA	This work
pBT-mcp2201	pBT derivative with λcl linked to the C-terminal region of MCP2201	This work
pBT-mcp2901	pBT derivative with λcl linked to the C-terminal region of MCP2901	This work
pBT-mcp2983	pBT derivative with λcl linked to the C-terminal region of MCP2983	This work
pBT-mcp3986(flmB)	pBT derivative with λcl linked to the C-terminal region of MCP3986	This work
pBT-mcp4715	pBT derivative with λcl linked to the C-terminal region of MCP4715	This work
pTRG	Bacterial two-hybrid bait plasmid with α-subunit of RNA polymerase (RNAp)	Stratagene
pTRG-cheA	pBT derivative with RNAp linked to the N-terminal region of CheA	This work
pTRG-flmA	pBT derivative with RNAp linked to the N-terminal region of FlmA	This work
pTRG-cheW	pBT derivative with RNAp linked to the N-terminal region of CheW	This work
pTRG-flmC	pBT derivative with RNAp linked to the N-terminal region of FlmC	This work

### Chemotaxis, twitching motility, and biofilm formation assays.

Chemotaxis assays were performed using semisoft agar plates with tryptone broth (TB) supplemented with 0.26% agar. Bacterial cells in logarithmic phase (OD_600_ of 0.4 to 0.7) from TB cultures were inoculated into the solidified agar and incubated at 30°C. Pictures were taken after 20 h of incubation. The twitching motility assay was performed as previously described (11). Briefly, colonies grown overnight on LB agar plates were picked using sterile toothpicks and stabbed into the bottom of petri dishes filled with medium and supplemented with 1% agar. Following incubation at 30°C in a humidified incubator for 24 h or 48 h, the agar and petri dish interface was inspected for a zone of motility. The biofilm formation assay was conducted by the method of O’Toole and Kolter (18) with slight modifications. Overnight cultures were diluted to an OD_600_ of 1.5, and 100 μl of the diluted sample was added to a 96-well PVC plate (Corning, MA, USA) as previously described (59). The plates were incubated at 30°C in a humidified incubator for 48 h. Planktonic cells were poured out carefully, and plates were washed with phosphate-buffered saline three times. One hundred twenty-five microliters of crystal violet (0.1%) was added to the wells and incubated for 30 min. After three washes, 150 μl of 30% acetic acid was added to dissolve the crystal violet, and the OD_590_ was measured on a multiwell plate reader. To determine whether the addition of ligands affects the growth of biofilm, bacteria were grown the same way as in the biofilm assay. Overnight cultures were diluted to an OD_600_ of 1.5, and the samples were divided into two equal parts. Ligands (final concentration, 2 mM) were added to one part, and the second part was left as a control. To determine growth, OD_600_ was measured in both samples. To compare growth of wild-type and mutant cells, 1% (vol/vol) bacteria from LB cultures were inoculated into minimal medium containing 2 mmol aromatic compounds as the sole carbon sources. A 200-ml mixture of bacteria and minimal medium were inoculated into each well of sterilized 100-well honeycomb plates, and the cell density at OD_600_ was monitored by using Bioscreen C automated growth curve analysis system.

### Confocal laser scanning microscopy (CLSM) and image acquisition.

In LB cultures, *C. testosteroni* CNB-1 and other strains grew to an OD_600_ of 2.0, and then bacteria were statically incubated at 30°C for 48 h. The air-liquid interface biofilms (pellicles) which grew in glass test tubes were moved onto glass slides. Biofilms were stained using SYTO9 and washed with phosphate buffer three times. Double-sided tape was used around the biofilm to maintain the gap between cover glass and biofilm. All ﬂuorescent images were acquired by a Leica SP8 (Leica Microsystems, Germany). CLSM-captured images were subjected to quantitative image analysis using COMSTAT software (60).

### Genetic cloning, overexpression, and protein purification.

Overexpression and purification of proteins were performed as previously described (42). Briefly, genes were cloned into pET28a to generate an N-terminal His-tagged fusion protein. Expression of the CheA gene was induced by the addition of 0.1 mM IPTG for 5 h at 30°C, while the expression of the response regulator genes (CheY_1_, CheY_2_, FlmD, and FlmE) and their mutants was induced at 16°C for 12 h. All proteins were then purified using AKTA FPLC equipped with a HisTrap HP column. Buffer desalting and protein concentration were performed by an Amicon Ultra-15 concentrator (Merck, MA, USA).

### Bacterial two-hybrid assay.

The BacterioMatch II Two-Hybrid system (Stratagene, CA, USA) was used to test the interaction between targeted proteins. Plasmid construction and screening were performed as previously described (61) and according to the manufacturer’s instructions. Briefly, overnight cultures were collected and washed by ddH_2_O three times. Bacteria (3 μl) were inoculated onto a selective screening medium plate containing 5 mM 3-amino-1,2,4-triazole (3-AT), 12.5 μg/ml streptomycin, 15 μg/ml tetracycline, and 25 μg/ml chloramphenicol to select positive growth cotransformants.

### Phosphotransfer assay.

All reactions were performed in TGMNKD (50 mM Tris-HCl, 10% [vol/vol] glycerol, 5 mM MgCl_2_, 150 mM NaCl, 50 mM KCl, 1 mM dithiothreitol [pH 8.0]) buffer at 25°C (62). To initiate the phosphorylation reaction, 10 μCi [γ-^32^P]ATP (PerkinElmer, MA, USA) was added to 100 μl of TGMNKD buffer that was previously mixed with 5 μM CheA. After 15 min, 10 μl of sample was taken (*T *=* *0) prior to the addition of any response regulators and quenched with 5 μl of SDS sample buffer. Then, the response regulators were added to mixtures to a final concentration of 10 μM. After specified time intervals, 10-μl samples were collected, and the reactions were stopped by the addition of 5 μl of sample buffer. The proteins were separated by 15% SDS-PAGE and exposed to a phosphorimaging screen. Quantitative analysis of the phosphotransfer efficiency was performed using Quantity One (Bio-Rad, CA, USA).

### Data sources, software, and analysis.

Sequences of chemotaxis proteins and associated information from *C. testosteroni* CNB-2 genome (identical to CNB-1 except for the loss of pCNB plasmid) were obtained from the MiST2.2 database (30). Multiple-sequence alignments were built using the L-IN-I algorithm from the MAFFT v4.182 package (63). Complete domain architectures for chemoreceptor sequences were obtained using the CDvist server (64). Chemoreceptors were assigned to heptad classes, and CheA, CheW, CheB, and CheR were assigned to evolutionary classes using previously described hidden Markov models (31, 35) and HMMER v.2.0 package (65). Methylation sites were identified from multiple-sequence alignment of the chemoreceptor signaling domain, using the consensus sequence [ASTG]-[ASTG]-x(2)-[EQ]-[EQ]-x(2)-[ASTG]-[ASTG] (35).
