# Supplementary figures and images for: Cross Talk between Chemosensory Pathways That Modulate Chemotaxis and Biofilm Formation
Source: mBio. 2019 Feb 26;10(1):e02876-18. doi: 10.1128/mBio.02876-18 (PMC6391922; doi:10.1128/mBio.02876-18)

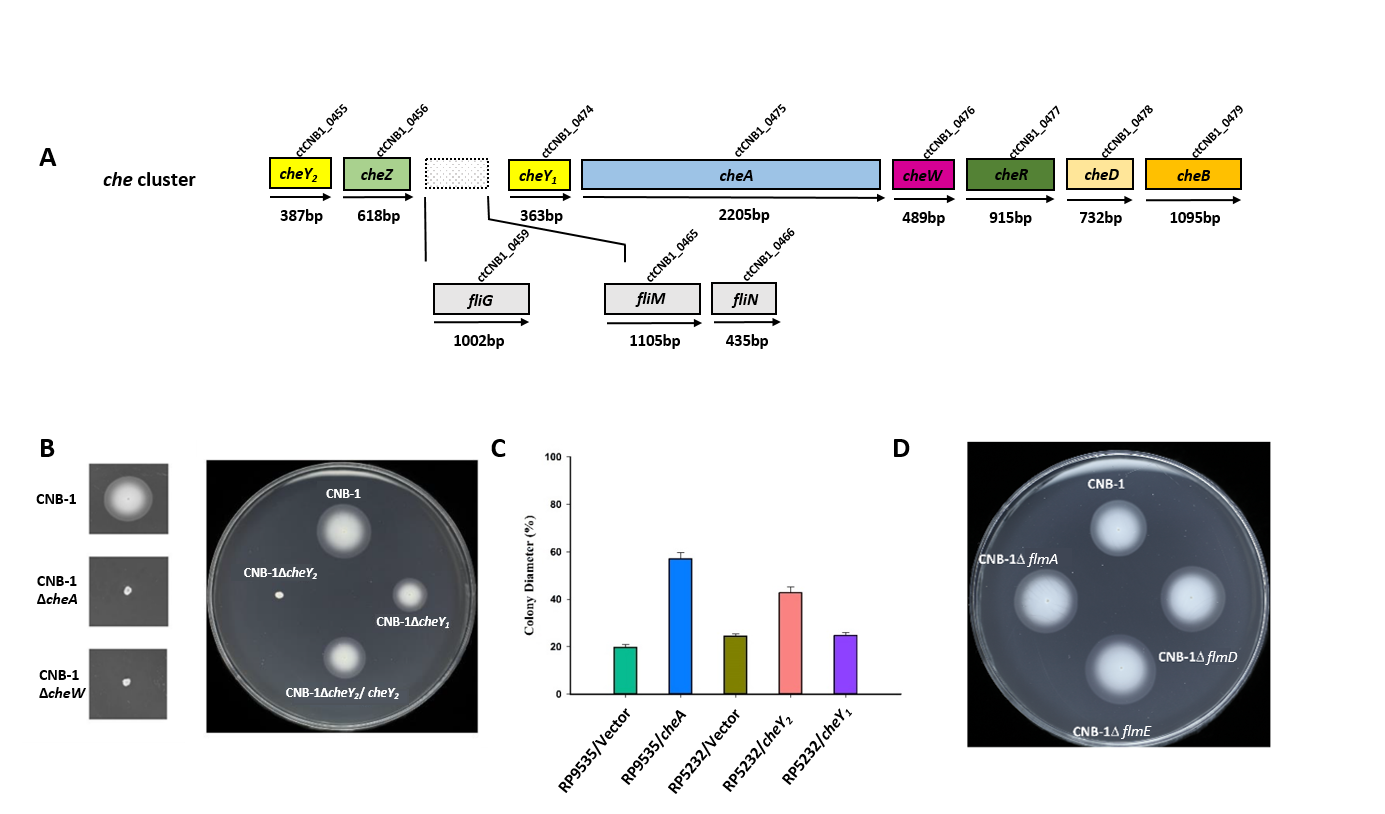

Supplement: FIG S1 [file mBio.02876-18-sf001.tif]

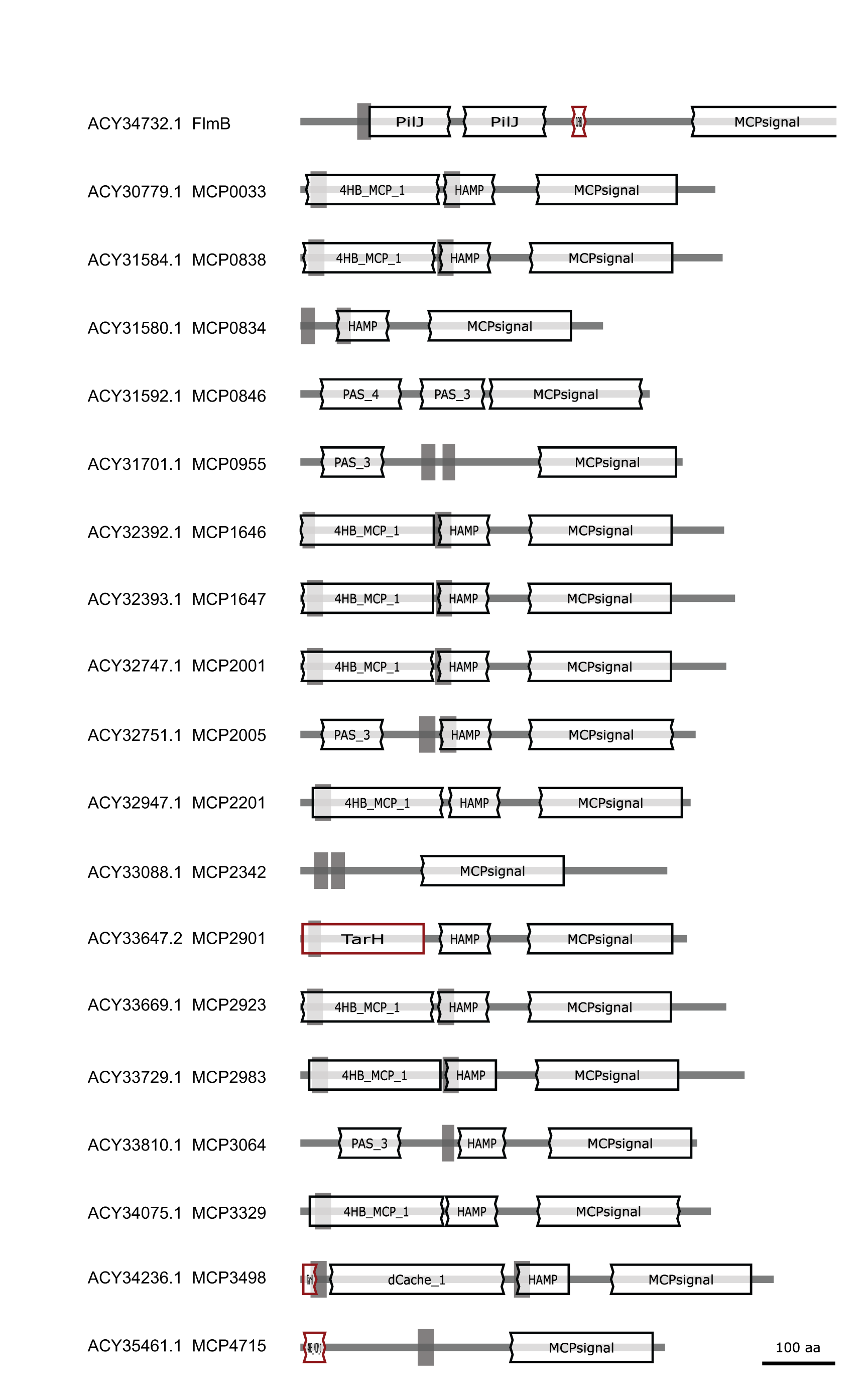

Supplement: FIG S2 [file mBio.02876-18-sf002.tif]

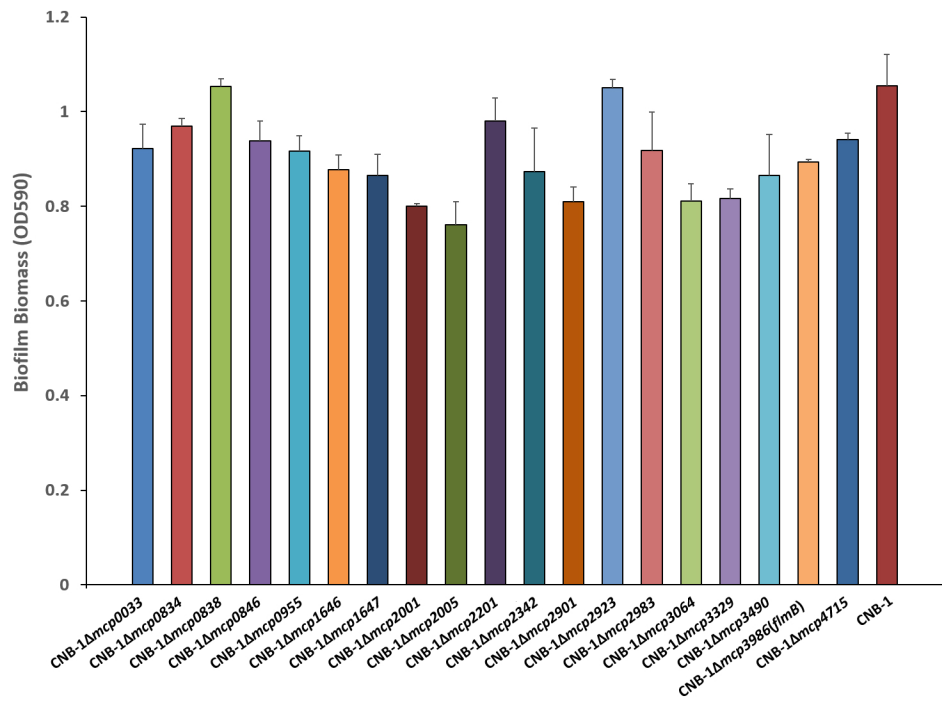

Supplement: FIG S4 [file mBio.02876-18-sf004.pdf]

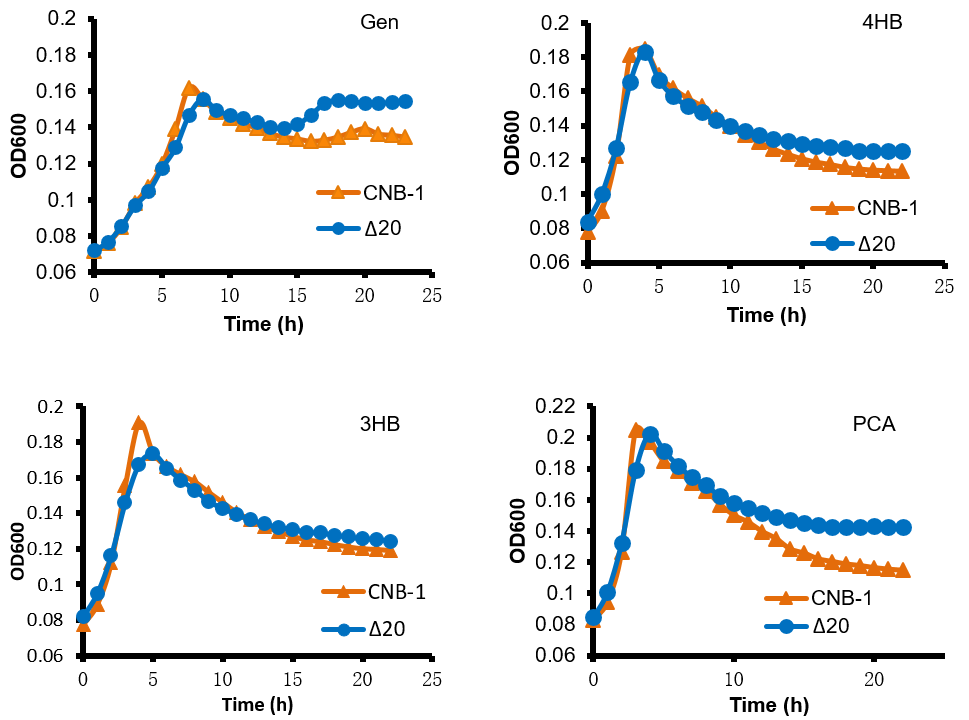

Supplement: FIG S5 [file mBio.02876-18-sf005.tif]

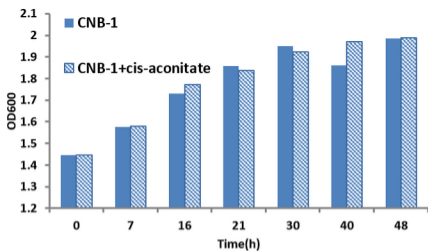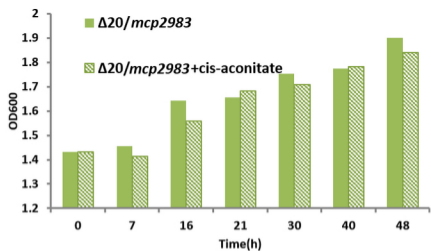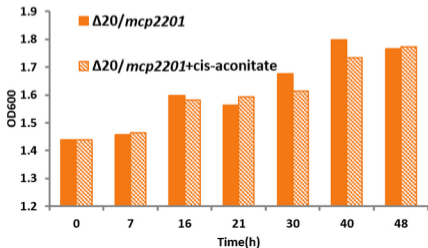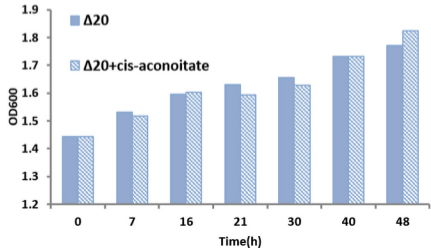

Supplement: FIG S6 [file mBio.02876-18-sf006.pdf]
