# Supplementary material for: Cross Talk between Chemosensory Pathways That Modulate Chemotaxis and Biofilm Formation
Source: mBio. 2019 Feb 26;10(1):e02876-18. doi: 10.1128/mBio.02876-18 (PMC6391922; doi:10.1128/mBio.02876-18)
Supplement: TABLE S1 [file mBio.02876-18-st001.pdf]

BLAST query			Best BLAST hit E values (% identity/% length coverage)				
			P. aeruginosa PAO1				
			NP_249099.1	NP_249100.1	NP_249101.1	NP_249102.1	NP_249104.1
			PilG	PilH	PilI	PilJ	ChpA
C. testosteroni	ACY34735.1	FlmE	5e-63 (63/85)				
	ACY34734.1	FlmD		9e-41 (51/95)			
	ACY34733.1	FlmC			6e-11 (27/92)		
	ACY34732.1	FlmB				4e-124 (38/89)	
	ACY34731.1	FlmA					2e-135 (38/74)
			C. testosteroni CNB-2				
			ACY34735.1	ACY34734.1	ACY34733.1	ACY34732.1	ACY34731.1
			FlmE	FlmD	FlmC	FlmB	FlmA
P. aeruginosa	NP_249099.1	PilG	2e-53 (63/85)				
	NP_249100.1	PilH		5e-41 (51/95)			
	NP_249101.1	PilI			3e-11 (25/88)		
	NP_249102.1	PilJ				2e-126 (38/95)	
	NP_249103.1	PilK			no significant hits		
	NP_249104.1	ChpA					6e-137 (38/70)
	NP_249105.1	ChpB			no significant hits		
	NP_249106.1	ChpC			no significant hits		
