# Supplementary material for: Cross Talk between Chemosensory Pathways That Modulate Chemotaxis and Biofilm Formation
Source: mBio. 2019 Feb 26;10(1):e02876-18. doi: 10.1128/mBio.02876-18 (PMC6391922; doi:10.1128/mBio.02876-18)
Supplement: TABLE S2 [file mBio.02876-18-st002.pdf]

Gene cluster	Chemotaxis class	Gene name	Accession	Function	M1 site	M2 site	M3 site	M4 site	Penta-peptide
che	ND	*cheY2*	ACY31201.1	response regulator					
	F7	*cheZ*	ACY31202.1	phosphatase					
	ND	*cheY1*	ACY31220.1	response regulator					
	F7	*cheA*	ACY31221.1	histidine kinase					
	F7	*cheW*	ACY31222.1	adaptor					
	F7	*cheR*	ACY31223.1	methyltransferase					
	ND	*cheD*	ACY31224.1	deamidase					
	F7	*cheB*	ACY31225.1	methylesterase					
flm	Tfp	*flmE*	ACY34735.1	response regulator					
	Tfp	*flmD*	ACY34734.1	response regulator					
	Tfp	*flmC*	ACY34733.1	adaptor					
	40H	*flmB*	ACY34732.1	MCP	no	no	no	no	no
	Tfp	*flmA*	ACY34731.1	histidine kinase					
orphans	36H	*MCP0033*	ACY30779.1	MCP	yes	yes	no	yes	no
	36H	*MCP0834*	ACY31580.1	MCP	yes	no	yes	no	no
	36H	*MCP0838*	ACY31584.1	MCP	yes	yes	yes	yes	yes
	ND	*MCP0846*	ACY31592.1	MCP	no	no	no	no	no
	36H	*MCP0955*	ACY31701.1	MCP	no	yes	yes	yes	no
	36H	*MCP1646*	ACY32392.1	MCP	yes	yes	yes	yes	yes
	36H	*MCP1647*	ACY32393.1	MCP	yes	yes	yes	yes	yes
	36H	*MCP2001*	ACY32747.1	MCP	yes	yes	yes	yes	yes
	36H	*MCP2005*	ACY32751.1	MCP	no	yes	yes	yes	no
	36H	*MCP2201*	ACY32947.1	MCP	yes	yes	yes	yes	no
	36H	*MCP2342*	ACY33088.1	MCP	no	no	yes	no	no
	36H	*MCP2901*	ACY33647.2	MCP	yes	yes	yes	yes	no
	36H	*MCP2923*	ACY33669.1	MCP	yes	yes	yes	yes	yes
	36H	*MCP2983*	ACY33729.1	MCP	yes	yes	yes	yes	yes
	36H	*MCP3064*	ACY33810.1	MCP	yes	no	yes	no	no
	36H	*MCP3329*	ACY34075.1	MCP	no	no	no	no	no
	36H	*MCP3498*	ACY3498.1	MCP	yes	yes	no	yes	no
	36H	*MCP4715*	ACY35461.1	MCP	yes	yes	yes	yes	no
