# Supplementary material for: Cross Talk between Chemosensory Pathways That Modulate Chemotaxis and Biofilm Formation
Source: mBio. 2019 Feb 26;10(1):e02876-18. doi: 10.1128/mBio.02876-18 (PMC6391922; doi:10.1128/mBio.02876-18)
Supplement: FIG S3 [file mBio.02876-18-sf003.pdf]

```
                                                                                           M1      M2     M3
ACY34732.1/432-747 QSVAEGDLT--QEATVTEDITGAIADSVNYTVEELRALVGSVQNTVTRVAQTTEQVDVTSTELLA-----ASNEQLHEIRETGKSILDMAGRIN---NVSAQAQESAQVARQSLQAA
ACY30779.1/232-562 LTIAGGDLS-HRAEVSGKDELTDLQRALDQMQQSLSTTVAQVRDASGNIATASQEIATGNQDLSA-----RTEQTASNLQETVASLAQLTATVQ---QTASSSQLANQLAASASSTA
ACY31580.1/87-411  ETVAAGDLS-KEFSSNAEGEFGRLLTALSAMEDTLTELVGRIKQSTDSLAVSAYEIDAGNINLSS-----RTEQQVSALTETAASMEQLTVTVR---QNAERAHSASSLAVTASATA
ACY32947.1/239-529 RRVADGDLT-ARIHVSSSDETGQLMQALHDMNTSLDRLVGQVRQGTDSIATASGQIAAGNHDLSA-----RTEEQASSLQQTAASMEQLTSTVK---QNADNASQANQLALSASDVA
ACY33729.1/237-602 QSVAAGDLS-ASITVQSQDEVGQLLQALKDMNTSLQRIVGQVRQGTDSIASASSQIASGNQDLSS-----RTEEQASSLEQTAASMEELTATVQ---QNAGNAQQANQMASAASQVA
ACY32392.1/225-574 QAVAAGRLG-MVVKNQRRDEMGLLLNALEDMRRQLSSVVQDVRGNAHGVALASSEIAQGNADLSA-----RTESQASALEETAASMEQLGSTVR---QNADNAQTANQMAKNASDVA
ACY32393.1/224-589 DRVANGDLS-GVIRVEREDETGRLLGALERMQSSLVQTVRSVRQNAEGVASASSQIASGNADLSG-----RTEEQASALEETAASMEQLGSTVR---QNADNARAANQMAMNASQVA
ACY32747.1/224-577 NRVASGDLS-ASISATSKDEMGLLLGALERMQHSLVTTVTSVRSNAQGVAAASAQIAAGNNDLSG-----RTEEQASALEQTAASMEQLGSTVK---QNAEHATQANQMAKSASVVA
ACY33669.1/224-577 DRVARGDLS-GQITVKSQDETGQLLSALQRMQHSLVSTVSAVRGNAQGVASGSAQIAMGNNDLSS-----RTEEQASALEQTAASMEQLSATVK---QNADNARQANQMAVNASSVA
ACY35461.1/202-494 RRVADGDLT----GGSSGGMARGLMSDLQTMRGALRELVQRVHGASVQMQHASSEIAQGNADLSA-----RTESQASALEETAASMEQLNATVR---QNADSAQTANRLAQNASQVA
ACY31584.1/226-572 DSIAAGDLT-ATVRNDRHDELGRLLKSLNAMAERLRSVVGQVRSGVESVSSASGQIATGNQDLSA-----RTEQTAANLEETAASMEELTATVT---QSADTARQANQLASTAAQAA
ACY33647.2/226-524 QAIAQGDLT-VTASNDRHDEFGRLLSSVSTMAAQLRSLVADVRTSVHSISTASAEIATGNHDLSA-----RTEQTAANLEETAASMEQLTATVA---QASDTARQANHLAGNAAQVA
ACY34075.1/235-557 QTIAAGDLSYAPPAAERGDELGRLMQSLALMTRQLRGLVGEVQGGVEAVAAASSQMAQDNSDLSD-----RTAHAAEQLKATVSSIENMVTLVT---HSADSARHADQSARSAAEAA
ACY31701.1/231-518 ADVAACNLS-TECSQSYSGAVGALMLRLQQIQINLRAVVGDVRTEVQGFAQTAHEIAQSSFDLAG-----RTESQATSLQQTAASMEEISGTVS---QTADTAQFMAGASDESSKVA
ACY32751.1/227-536 NQLASGDLS-HVVRTGASGSIGNLQRALFQLSVNLRTVVSDVRQEVGHLEIAVREIASGNQSLSS-----RTETQASSLEQTAASMEQINGTVH---NSALSASRGARFAEEASGIT
ACY33810.1/252-538 LNVATGNNR-QTEYLQRSDEIGTTLRTVNQIGLMFRWLVDDVNQQALNVQQACNEIEQGNSYLHG-----QTEQLAVNVAQTSASMEQITARVQ---SSADTAQKAGVLASEASAAA
ACY33088.1/78-497  EKVAHGDLS--MSAAKGAQAAAATSGLTESMVHALSAMVADVRSNAALVAQAGQGMVKDHQALSE-----RTEMQASNLAQTVVSVEQLSAAVQ---HSAEVSKAAHGRADGVRSAA
ACY34236.1/334-641 IADGHGDLS-RRMPVESNDEVGALASAFNRFVSSLERMVGAVRQAADSISVASSEVAQGNQDLSQ-----RTEQAASALQQTAASLSVLTDSVQ---ANTEATRSAGELAQSARGVA
ACY31592.1/208-474 HKLALGHHD-----------AGQYLR------------------IGKAGRQVWIE-ASYNPILDAEGRPFKVVKFATNITRRVTAAQTLRATVEGLTESANHARQANELAQDACSVA
Con_90%            .tlAtGphs........tt.hs.h.t.h...h...h..hVttlptts..ht.sstphs.ss..Lts.....potp.sttltpTssShtphsstVp...psutpup.usthA.tuttsA
Con_60%            ppVAsGDLo.tphpspppDEhGpLhpuLppMppsLpslVupVRssspulApASspIAsGNpDLSu.....RTEpQASsLcpTAASMEQLsuTVp...QsA-sAppANphApsASplA
                                                         N22    N21    N20    N19        N16    N15    N14    N13       N12    N11    N10
                                                         abcdefgabcdefgabcdefgabcdef-----gabcdefgabcdefgabcdefgab---cdefgabcdefgabcdefga

ACY34732.1/432-747 DSGLKAVQNAIGGMNSIRDQIQDTSKRIKRLGESSQEIGEITELISDITEQTNVLALNAAIQAASAGEAGRGFSVVAEEVQRLAERSADATRQIAALVKAIQTDTQDAVAAMERSTQGVV
ACY30779.1/232-562 VQGGEIVGQAVNSMQEISASSRKIGDI--------------IGLIDSIAFQTNILALNAAVEAARAGEQGRGFAVVAAEVRSLAQRSAQAANEIKSL--------------ISTSVQTVD
ACY31580.1/87-411  GRGGDVVDQVVHTMDAISGSSRKIVDI--------------IQVIEGIAFQTNILALNAAVEAARAGEQGRGFAVVASEVRSLAQRSAEAAKQIKEL--------------ITASVTQVE
ACY32947.1/239-529 VKGGMVVSQVVETMGAISQSSRKISDI--------------IGVIDSIAFQTNILALNAAVEAARAGEQGRGFAVVASEVRSLAGRSAEAAKEIKQL--------------IQASVTKVE
ACY33729.1/237-602 VRGGATVAQVVQTMSAINDSSRKIVDI--------------IGVIDSIAFQTNILALNAAVEAARAGDQGRGFAVVASEVRTLAQRSAEAAKQIKQL--------------IDDSVSKVQ
ACY32392.1/225-574 GRGGDVVAQVVDTMKGINDSSRQIADI--------------IGVIDSIAFQTNILALNAAVEAARAGEQGRGFAVVAGEVRTLAQRSAEAAKEIKQL--------------IHASVERVE
ACY32393.1/224-589 AQGGAVVAEVVETMKGINNSSQQIADI--------------ITVIDSIAFQTNILALNAAVEAARAGEQGRGFAVVAGEVRTLAQRSAEAAKEIKAL--------------ISTSVQRVE
ACY32747.1/224-577 IEGGKVVDQVVATMKSIDESSNKIADI--------------ISVIDSIAFQTNILALNAAVEAARAGEQGRGFAVVASEVRALAGRSAEAAKEIKTL--------------ITTSVQRVE
ACY33669.1/224-577 TQGGEVVAEVVETMKSINESSHKISDI--------------IAVIDGIAFQTNILALNAAVEAARAGEQGRGFAVVASEVRALAGRSAEAAKEIKQL--------------ITTSVQRVE
ACY35461.1/202-494 RQGGAVVARVVQTMQDINTSSSRIADI--------------IGVIDAIAFQTNILALNAAVEAARAGEQGRGFAVVASEVRALAGRSADAAKEIKML--------------ITASVERVA
ACY31584.1/226-572 EQGGRVVQQVVQSMGQITDSSRKIADI--------------IGVIDSIAFQTNILALNAAVEAARAGEQGRGFAVVAGEVRSLAQRSAEAAKEIKQL--------------ITTSVDNVQ
ACY33647.2/226-524 QRGGHVVQSVVSSMGRISDSAHRISDI--------------IGVIDSIAFQTNILALNAAVEAARAGEQGRGFAVVAGEVRALAHRSAEAAKEIKAL--------------ITTSVDAAQ
ACY34075.1/235-557 ASGGSVVQEVVRNMEHMAQSSQQVAAI--------------VGVIDGIAFQTNILALNAAVEAARAGTQGRGFAVVAAEVRELAQRSAEAAKQIRQL--------------MEHSTKQMK
ACY31701.1/231-518 SRSGAAISEVGLAMEHIRGSSTRMSEI--------------IGVIESIAFQTNLLALNAAVEAARAGEQGRGFAVVAGEVRALAQRSAEAAKEISVL--------------INRTVDGIN
ACY32751.1/227-536 QRSNDAVQAVGRTMLGIAESSRHIEDI--------------VHLIEGVAFQTNILALNAAVEAARAGEAGRGFAVVASEVRTLSARTTQAARDIKQL--------------IAESAERVA
ACY33810.1/252-538 LRGGQSMDQIVSTMESITANSRRIADI--------------VGVIDSMPFQTNLLALNAAVEAARAGEHGRGFAVVAGEVRALAQRSADAAKEIKAL--------------IQASTATIE
ACY33088.1/78-497  DQGAQAMQHAVQSVELIERNAGRMREI--------------IGVIDGIAFQTNILALNAAVEAARAGEQGRGFAVVASEVRTLAQRSAEAAKEIRQL--------------IGETVQQVS
ACY34236.1/334-641 ERGGTAFQQVVVTMEGINHSSRKIADI--------------IGVIDSIAFQTNILALNAAVEAARAGEQGRGFSVVAAEVRSLAQRSAEAAKEVRQL--------------ITDSVTQVG
ACY31592.1/208-474 EAGGRTVQDVVTTMESISASSRKISDI--------------IGMMDSIAFQTNLLALNAAVEAARAGDQGRGFAVVANEVRQLAQHSAEAAKEIKQL--------------IQTSVTQVT
Con_90%            .pustshtpsstsMttIttsupph.-I..............ltlI-ulsFQTNlLALNAAVEAARAG-tGRGFAVVAsEVRtLAtRSApAA+pIp.L..............lttosptht
Con_60%            spGGplVspVVpTMpuIssSS++IuDI..............IGVIDSIAFQTNILALNAAVEAARAGEQGRGFAVVAuEVRsLAQRSAEAAKEIKpL..............IssSVppVp
                         N09    N08    N07    N06    N05    N04    N03    N02    N01    C01    C02    C03    C04    C05    C06    C07    C08
                   bcdefgabcdefgabcdefgabcdefgabcdefgabcdefgabcdefgabcdefgabcdefgabcdefgabcdefgabcdefgabcdefgabcdefgabcdefgabcdefgab

                                                                                  M4
ACY34732.1/432-747 EGARLSDSAGTALSEIDSVSRRLAELIEQISNSTSREANMANGVAENIQHIFAVTEQTGEGTRTTAQQVRELSHMAEELRQSVSRFKIA-------------------------------
ACY30779.1/232-562 VGARQVESAGKAMQETVDSAQRVGDIIGEITAASSEQSLGIGQVNQAVGDIDRMTQQNAALVEESAAAAESLREQAARLAQLVSQFKLAGGA--------MHAVMRS----PRGAAHPGM
ACY31580.1/87-411  SGSGLVGQAGSTMKEIMQSVGQVTGLLSEISGALQQQSEGIAHVNTAVAHMDSTNQENAALVMQATQAAAALNARTQDLQQAVGAFKLDDDE----SPCLAPSAMPA----PRGAG----
ACY32947.1/239-529 EGSAQVSQAGQTMDEIVSSVQRVTDIMGEITAASHEQTSGIEQINRAVAEMDLVTQQNAALVEESTAAAQSMQQQTSDLSQMVSVFRLKSA-----------------------------
ACY33729.1/237-602 EGSTQVDEAGRTMDEIVMSVRRVTDIMGEISVASHQQTSGIEQVTQAVTQMDQMTQQNAALVEEAAAATDALRSQAAALAETVSFFKLGAQSGLQATPALAPAALKS---APARAAIK--
ACY32392.1/225-574 QGSQLVDKAGDTMAEIVTAIGRVTDIMGEISAASREQSQGVAQVGEAVTQMDQTTQQNAALVEQSAAAADSLQRQAKALVDSVAIFQLGNQA-----QSFAAAALKNRV-EPVAKAGM--
ACY32393.1/224-589 QGTQLVDKAGATMADIVSAISRVTDLMAEISAASQEQSQGVAQVGEAVTQMDQTTQQNAALVEESAAAAGALRKQAQDLVQAVAVFQLPASA---LYDQAPKAAARAAA--PAVVAAAAP
ACY32747.1/224-577 QGSLLVDKAGRTMTDVVSAIGRVTDIMGEISAASQEQSQGVAQVGEAVTQMDQTTQQNAALVEEMAAAASSLNHQARSLVEAVAVFTLSEQI------QGPDSGM------PTTSASVTK
ACY33669.1/224-577 QGSLLVDKAGETMTGVVTAIRRVTDIMGEISAASHEQSSGMAQIGEAVTQMDQTTQQNAALVEEMAAAASSLHNQAQSLVEAVAVFKLSEHE------QHASAAMQAASVNPTKTATVAK
ACY35461.1/202-494 DGSALADQAGRTMDEIVSAIHRVTDIMGEISAASSEQSAGVAQVGEAVMQMDQATQQNAALVEESAAAAEGLRQQAESLLTEVSRFQLERGT----------LALR--------------
ACY31584.1/226-572 SGSQQVELAGQSMSEIVASVRRVSDLIGEITASSTEQRDGINQVNQAVSNLDQMTQQNTALVEESSAAALSMQEQARRLAEVVSVFKLGRNA---EAFTTVSAVLPK----PLATIKALN
ACY33647.2/226-524 EGADQVSQAGRVMEEIVSSVGKVSDMIGEISASAMEQHDGISQVNQAVTNLDQMTQQNAALVEESTAAAVSLSDQAQRLTGMVSVFK-------------VQAADRA----PALTV----
ACY34075.1/235-557 SGHTLALQAGQRMEHIVRDVRTVSGLIASITEAAQAQNQGIAQISEAVQALDHMTSQNAGLVAESSAAARELFHQAQRLEAGAGRFRIDYQALDNEIADIEVDVMKREVHVPRLAALEA-
ACY31701.1/231-518 DGNARMRAAGQTIDGMVQAVDKVGSLVHQISIATREQSIGISQVNEAVAQLDSVTQQNAALVEQSTSAAQSLRLSARTLERSVDVFHL--------------------------------
ACY32751.1/227-536 SGASATGEARERMTEALTAVQRVSVVLGEISTAAVEQQTGISQVNAAVAHMDSITQQNVAMVEELAAAAQSLRDQVHGVSSSMRLFRLERGE----------------------------
ACY33810.1/252-538 AGSLVVSDAGSNIGDIVSQARSVADFIAEISKATREQSEEISQVGKSVADMDYLTQQNAQLVTQGAQASSSMQWQVKNLVEAIGVFR---------------------------------
ACY33088.1/78-497  QSTQLLRNAVEQTQGVTRAIHDVVSHMWTLSDSASSQSTGLQEISNAVHQIDEITQHNAQMVGQALQQARELQLRAHQLSDAVSRFRLQQGT-AEEAVALVQATMRQAK-SSSSVASLLQ
ACY34236.1/334-641 SGSQQVEHAGATMQELLDAVARVTRIIEEISDASQTQGRGIAEINQSVAGLDDATQQNSALVEQSAAAATSLREQAARLMGEVSAFKLGEVPQRSPFAQEQQQQLLEA
ACY31592.1/208-474 QGVNQVREAGTTMAEIVNSSRQVTQIMGEVVQTSLAQSARLREVT------DDLTAQDVSAHPGSVRPRPAVPLQASAREQATIAIRNAAVG----------------------------
Con_90%            tGs..httAspthtthhts.tpVsthhtplo.us.tQp.thtplstsl.thD.hTppssths.p.stts.th..psttl.t.hthFp.................................
Con_60%            pGophVcpAGpoMsEIVsulpRVoDlhGEISsAopEQSpGluQVspAVsphDphTQQNAALVEEouAAApuLppQApsLspuVuhF+Lsttt..........ssh......s...h....
                        C09    C10    C11    C12    C13    C14    C15    C16   C19    C20    C21    C22
                   cdefgabcdefgabcdefgabcdefgabcdefgabcdefgabcdefgabcdefgabcdefgabcdefgabcdefgabcdefgabcdefg

ACY34732.1/432-747 -------------------------------------------------------------------------------------------------------
ACY30779.1/232-562 APAVPA-------------------SAQAALPGQKA--------------------------QLLEHA-----------------------------------
ACY31580.1/87-411  --------------------------AAQARP-------------------------ARARELAYEEL-----------------------------------
ACY32947.1/239-529 -------------------------------------------------------------------------------------------------------
ACY33729.1/237-602 -AAAPLKA----------------PSAALKRPAMSVGSKPPVLSPAKSAAAPQPQKAATADDGEWESF-----------------------------------
ACY32392.1/225-574 -SAGPRAA-----------------ALAGQGPGK------------AVPAPVKTAQPALSNDQDWEQF-----------------------------------
ACY32393.1/224-589 ARQAPVRR-----------------VAARPAPMPAAPRSLMGGAVAGESATARNVQKSSLDEDDWETF-----------------------------------
ACY32747.1/224-577 GTLAPN----------------------LSKPLQASLASIKSTVERRPASIEGTQRSTGKTDVDWETF-----------------------------------
ACY33669.1/224-577 VSESP------------------------PKPLMAT----KSPVLRRPVAIAGTQQATNKNGDDWETF-----------------------------------
ACY35461.1/202-494 -------------------------------------------------------------------------------------------------------
ACY31584.1/226-572 PAAQPA--------------------VVAPRP--------------VASRSKEQSLASTAEDDSWEQF-----------------------------------
ACY33647.2/226-524 -------------------------------------------------------------------------------------------------------
ACY34075.1/235-557 -----------------------------------------------------------------EMA-----------------------------------
ACY31701.1/231-518 -------------------------------------------------------------------------------------------------------
ACY32751.1/227-536 ------------------------------VPL-------------SQASAVELRRTSRLR------------------------------------------
ACY33810.1/252-538 -------------------------------------------------------------------------------------------------------
ACY33088.1/78-497  TVTDPAQPFHDRDMYVFALSSEGEYRAFGGNPAKVGLRVQDVPGIDGDSLMADIMAQAELRPGWVEYDYINPSNQQVQTKMSYVCKHAGLYWGCGVYKSLVSV
ACY34236.1/334-641 -------------------------------------------------------------------------------------------------------
ACY31592.1/208-474 -----------------------------------------------------------------AY------------------------------------
```
